# Activation of the nuclear factor E2-related factor 2/anitioxidant response element alleviates the nitroglycerin-induced hyperalgesia in rats

**DOI:** 10.1186/s10194-016-0694-x

**Published:** 2016-10-24

**Authors:** Wei Di, Xiaolei Shi, Hua Lv, Jun Liu, Hong Zhang, Zhiwei Li, Yannan Fang

**Affiliations:** 1 Department of Neurology, Shaanxi Provincial People’s Hospital, The Third Affiliated Hospital of Xi’an Jiaotong University, Shaanxi, 710068 China; 2 Department of Neurology, The First Affiliated Hospital, Yijishan Hospital of Wannan Medical College, Anhui, 241001 China; 3 Guangdong Key Laboratory for Diagnosis and Treatment of Major Neurological Diseases, Department of Neurology, National Key Clinical Department and Key Discipline of Neurology, The First Affiliated Hospital, Sun Yat-sen University, Guangdong, 510080 China

**Keywords:** Migraine, Nitroglycerin, Hyperalgesia, Nrf2/ARE, Trigeminovascular system

## Abstract

**Background:**

Antioxidants have been proven to weaken hyperalgesia in neuropathic pain. Endogenous antioxidant defense system may have a role in the prevention of hyperalgesia in migraine. In this study, we aimed to evaluate the role of nuclear factor E2-related factor 2/antioxidant response element (Nrf2/ARE) pathway in regulating the activation of the trigeminovascular system (TGVS) and hypersensitivity in nitroglycerin (NTG)-induced hyperalgesia rats.

**Methods:**

The expression levels of Nrf2, HO, HO1, and NQO1 in the trigeminal nucleus caudalis (TNC) were detected by western blot. Immunofluorescence was used to demonstrate the cell-specific localization of Nrf2 in TNC. Sulforaphane, a Nrf2 activator, was administered to NTG-induced rats. Then, the number of c-Fos- and nNOS-immunoreactive neurons in TNC was evaluated using immunofluorescence, and c-Fos and nNOS protein levels were quantified using western blot. Von Frey hair testing was used to evaluate the tactile thresholds of rats at different time points in different groups.

**Results:**

Total cellular and nuclear levels of the proteins Nrf2, HO1, and NQO1 were elevated in TNC after NTG injection, and Nrf2 was found to be located in the nucleus and cytoplasm of the neurons. Sulforaphane pretreatment significantly increased the nuclear Nrf2, HO1, and NQO1 levels in TNC. In addition, sulforaphane exposure effectively inhibited the expression of nNOS and c-Fos, reduced the number of nNOS and c-Fos immunoreactive neurons in TNC, and attenuated the tactile thresholds induced by NTG injection.

**Conclusion:**

Oxidative stress was involved in nitroglycerin-induced hyperalgesia. Activation of the Nrf2/ARE pathway inhibited the activation of TGVS and prevented the induction of hyperalgesia. Sulforaphane might therefore be an effective agent for hyperalgesia. Further studies are needed to discover the underlying mechanisms of the process.

## Background

Migraine is a primary headache disorder characterized by recurring, episodic, and unilateral throbbing pain in the head. Cutaneous hyperalgesia occurs during migraine attacks, and is a risk factor for the recurrent attack and chronification of migraine [1, 2]. Exploring the potential mechanisms of this feature may warrant preventive treatment strategies.

Nitroglycerin (NTG) is widely used to create a validated animal model of migraine for further exploration of the pathogenesis and treatment of the disorder [3, 4]. Systemic administration of NTG causes a delayed spontaneous headache attack and induces the consequent central sensitization in migraineurs or rats via an NO-dependent pathway [5–7]. And trigeminovascular system (TGVS) activation participates in the nociceptive transmission, thereby enhancing this chronic sensitization process [8].

Several studies have shown that oxidative stress plays a role in central sensitization [9, 10]. Reactive oxygen species scavenger alleviates hyperalgesia in rats with neuropathic pain, suggesting that potential role of antioxidants [11, 12]. It has been found that the nuclear factor E2-related factor 2/antioxidant response element (Nrf2/ARE) pathway is the most important endogenous antioxidant defense system, and plays a critical role in regulating cellular oxidation, cell defense, and protection [13]. Increasing data points out the protective role of Nrf2/ARE pathway activation in the brain [14]. However, the role of Nrf2/ARE pathway in hyperalgesia in migraine remains unclear.

Thus, the aim of this study was to investigate the role of Nrf2/ARE pathway in NTG-induced hyperalgesia and its underlying mechanism. By doing so, we expect to find an effective therapeutic approach for this disorder.

## Methods

### Animals

Male Sprague-Dawley rats (*n* = 132, weight 180-220 g) obtained from the Laboratory Animal Center of Sun Yat-sen University (Guangzhou, China) were used for the study. The animals were housed in groups of 3–4 with water and food available ad libitum and were kept under a 12-h light/dark cycle at constant temperature (25 ± 1 °C) conditions. All experiments were conducted according to the international association for the study of pain (IASP) guideline and every effort was made to minimize animal suffering.

### Drug administration

NTG (Beijing Yimin Pharmaceutical Co., Ltd, China) was injected subcutaneously (s.c) in the back of rats (10 mg/kg) from a stock of 5.0 mg/ml. Control rats were subcutaneously injected with an equal volume of 0.9 % normal saline (NS) as a vehicle for NTG [3].

R, S-Sulforaphane (SFN) (LKT Laboratories, Inc., St. Paul, MN) was dissolved in sterilized distilled water according to the instructions, and a dose of 5 mg/kg was administered intraperitoneally (i.p) based on previous studies [15, 16]. The vehicle group was also injected intraperitoneally with an equal volume of sterilized water.

### Experimental protocol

First, 60 rats were randomly separated into ten groups according to the different time points (0, 0.5 h, 1 h, 2 h, and 4 h) after NTG/NS injection. TNC tissue samples of the rats were taken for analyzing the expression levels of total cell Nrf2, nuclear Nrf2, HO, and NQO1 using western blot. A group of 18 rats was used to demonstrate the cell localization of Nrf2 in TNC among the groups (Control, NTG 2 h, and NTG 4 h) by immunofluorescence. Second, rats were divided into five groups as follows: 1) Control group (*n* = 6), rats received NS (s.c) in a volume equal to that of NTG, 2) SFN plus control group (*n* = 6), rats received SFN (5 mg/kg i.p) 30 min before NS (s.c), 3) NTG group (*n* = 6), rats received NTG (10 mg/kg s.c), 4) H_2_O plus NTG group (*n* = 6), rats received sterilized distilled water (i.p) 30 min before NTG (10 mg/kg s.c), and 5) SFN plus NTG group (*n* = 6), rats received SFN (5 mg/kg i.p) 30 min before NTG (10 mg/kg, s.c). Von Frey hair testing was used to evaluate the tactile sensitivity threshold. Western blot was used to detect the c-Fos, nNOS, nuclear Nrf2, HO1, and NQO1 expressions in TNC. Finally, rats were divided into four groups as follows: 1) Control group (*n* = 6), rats received a subcutaneous injection of NS (s.c) in a volume equal to that of NTG, 2) NTG group (*n* = 6), rats received NTG (10 mg/kg s.c), 3) H_2_O plus NTG group (*n* = 6), rats received sterilized distilled water (i.p) 30 min before NTG (10 mg/kg, s.c), and 4) SFN plus NTG group (*n* = 6), rats received SFN (5 mg/kg i.p) 30 min before NTG (10 mg/kg, s.c). Immunofluorescence was performed to evaluate the numbers of c-Fos and nNOS-immunoreactive neurons in TNC.

### Behavior test

Tactile sensitivity threshold was evaluated with calibrated (0.008 g, 0.02 g, 0.04 g, 0.07 g, 0.4 g, 0.6 g, 1.0 g, 1.4 g, 2.0 g, 4.0 g, 6.0 g, 8.0 g, 10.0 g, and 15.0 g) von Frey hairs (Stoelting Co., Wood Dale, Illinois, USA) by the up-down method as described previously [17, 18]. Briefly, the rats were accommodated in the testing chambers for a period of 30 min prior to the testing. A series of von Frey hairs with logarithmically incremental stiffness was applied to the periorbital region of the face and middle of the plantar surface of the front paw for 6-8 s at intervals of 30 s between consecutive stimuli. Quick withdrawal or licking of front paw in response to the stimulus or scratching of the periorbital region in response to the stimulus was considered as a positive response. The tactile thresholds to the stimuli of von Frey hairs were analyzed at baseline, 0.5, 1, 2, 3, and 4 h after NTG or NS injection by experimenters who were blinded to each rat group.

### Immunofluorescence staining

Rats were anaesthetized with 10 % chloral hydrate (3 ml/kg, i.p) and then perfused transcardially with 0.9 % saline at 4 °C followed by 4 % paraformaldehyde in phosphate buffered saline (PBS 0.1 mol/L, pH 7.4). Regions from the medulla oblongata to the first cervical cord were immediately isolated, fixed in 4 % paraformaldehyde, cryoprotected in 30 % sucrose, frozen, and serially sectioned 1–5 mm from obex (10 μm-thick transverse sections) on a cryostat (CM1900, Leica, Heidelberg, Germany). Sections were incubated with primary antibody against Nrf2 (rabbit polyclonal antibody, dilution 1:200, Abcam, UK), neuronal nuclei NeuN (mouse monoclonal antibody, dilution 1:500, Millipore, USA), c-Fos (rabbit monoclonal antibody, dilution 1:200, Abcam, UK), and nNOS (rabbit monoclonal antibody, dilution 1:200, CST, USA). After rinsing in PBS (0.01 mol/L, pH 7.4), sections were incubated for 1 h at 25 °C with secondary antibody, Alexa Fluor 488-conjugated anti-rabbit IgG (1:200, Jackson, USA) or Dylight 549-conjugated anti-mouse IgG (1:200, Jackson, USA). Sections were mounted in fluorescent mounting medium (R&D systems, Minneapolis, MN, USA), and signals were detected using a fluorescence microscope (BX51, Olympus, Japan). Negative control sections were incubated with PBS instead of primary antibodies and they showed no positive signals. The number of c-Fos- and nNOS-immunoreactive neurons in TNC confined to a 400 × 300 μm square was determined using Image J software (version 1.4.3.67, NIH). Data from 10 regions sampled from each section (10 sections per rat) were averaged and presented as number per 1.2 × 10^5^ μm^2^ in TNC.

### Immunoblotting

After anaesthetizing as described above, the TNC was rapidly dissected, 1–5 mm from obex, homogenized in tissue lysates (Pierce, Rockford, IL, USA) with protease inhibitor cocktail (Merck, Darmstadt, Germany), and the protein concentration was determined using BCA reagent (Pierce, Rockford, IL, USA). Equal amounts of protein extracts were separated electrophoretically on 10 % sodium dodecyl sulfate-polyacrylamide gels and transferred onto polyvinylidene difluoride membranes (Millipore, Temecula, CA, USA). After blocking with 5 % skimmed milk for 1 h, the membranes were incubated with primary antibody against Nrf2 (rabbit polyclonal antibody, dilution 1:1000, Abcam, UK), HO1 (rabbit monoclonal antibody, dilution 1:1000, Abcam, UK), NQO1 (rabbit polyclonal antibody, dilution 1:1000, Abcam, UK), c-Fos (rabbit monoclonal antibody, dilution 1:500, Abcam, UK), nNOS (rabbit monoclonal antibody, dilution 1:1000, CST, USA), β-actin (mouse monoclonal antibody, dilution 1:5000, Sigma, USA), and fibrillarin (mouse monoclonal antibody, dilution 1:4000, Abcam, UK). For the negative control, rabbit primary antibody was replaced by normal serum. This was followed by adding horseradish peroxidase-conjugated anti-mouse or anti-rabbit secondary antibody (dilution 1:5000, Jackson, USA). An enhanced chemiluminescence kit (Millipore, Temecula, USA) was used for the visualization of the bands. Densitometric analysis was performed using Image J software.

### Statistical analysis

All data are expressed as the mean ± SD. Statistical analyses were performed using IBM SPSS version 17.0 (SPSS Inc., Chicago, IL, USA). Data at different time points were analyzed using a two-way analysis of variance (ANOVA) followed by Bonferroni post-hoc-test. Other data were analyzed using a one-way ANOVA followed by Bonferroni post-hoc-test. *P* value < 0.05 was considered as statistically significant.

## Results

### NTG altered the antioxidant system in TNC

**Fig. 1:**
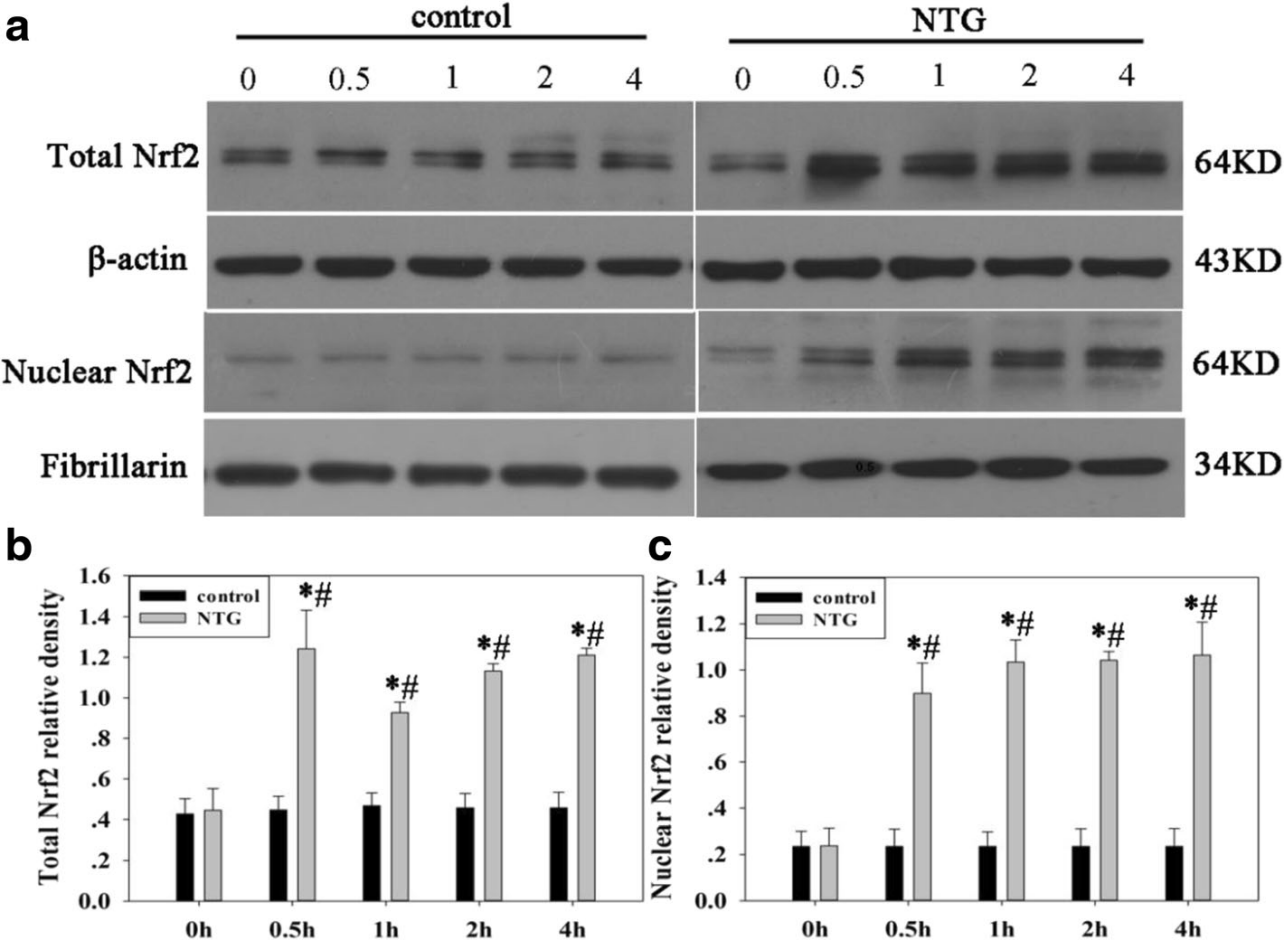
Effect of NTG injection on Nrf2 protein levels in the total and nuclear fractions of rat TNC **a** Representative immunoblots of TNC lysates. Total Nrf2 levels **b** and nuclear Nrf2 levels **c** were elevated as early as 0.5 h and persisted for 4 h after NTG injection. β-actin was used as a loading control for total Nrf2. Fibrillarin was used to assess the purity of the nuclear fraction. Data are presented as relative density units normalized to β-actin or Fibrillarin, and expressed as mean ± SD (*P < 0.01 *vs* the control group, # *P* < 0.01 *vs* NTG 0 h group, *n* = 6 per group)

**Fig. 2:**
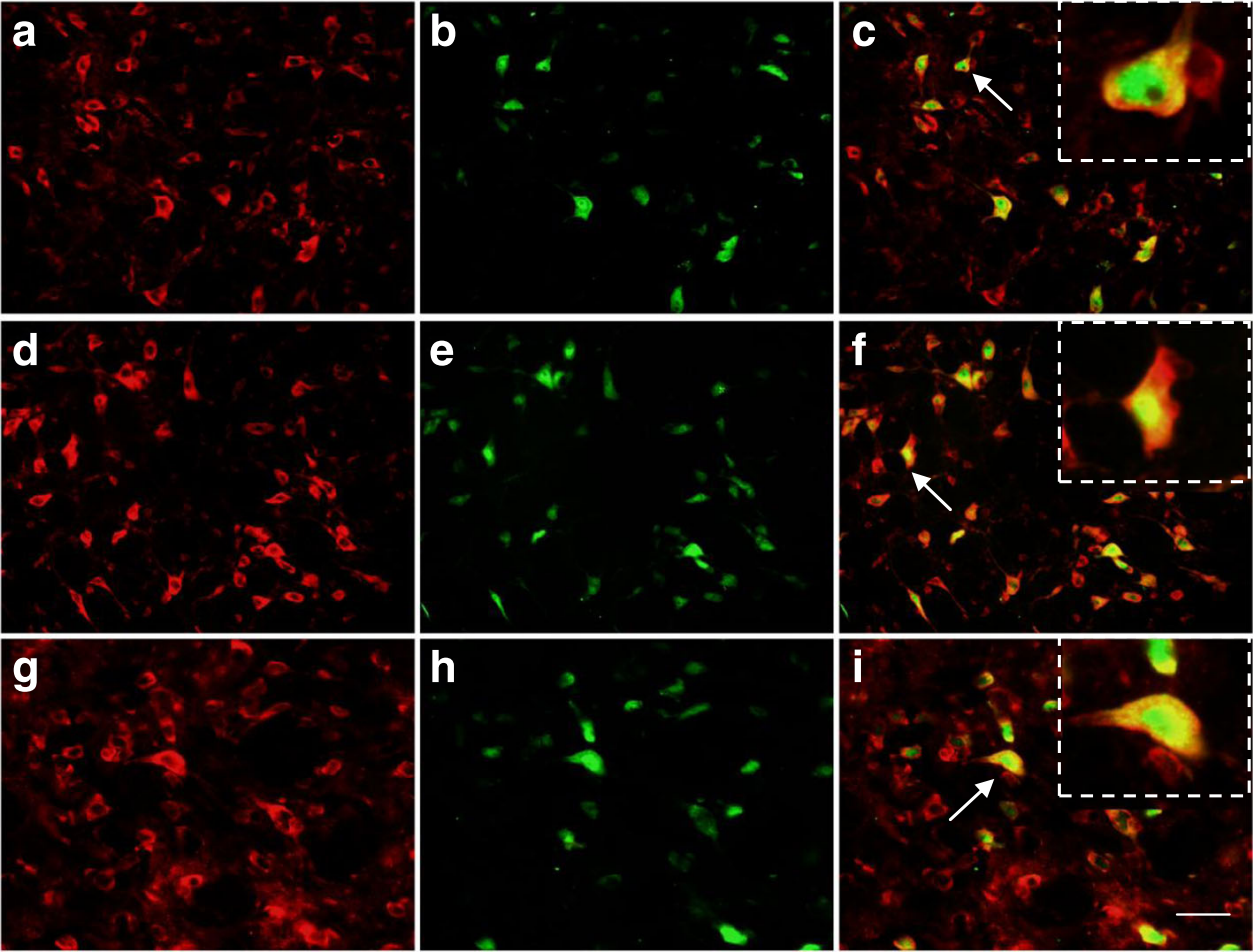
Co-localization images of Nrf2 with NeuN in TNC. Red indicates Nrf2 immunoreactivity, green indicates NeuN immunoreactivity, and yellow indicates merged signal. In control group **a**-**c** of the TNC, Nrf2 is present mainly in the cytoplasm (shown by arrows). In the NTG 2 h group **d**-**f** and NTG 4 h group **g**-**i**, Nrf2 staining was observed both in the cytoplasm and in the nucleus (shown by arrows). Bar = 50 μm. The arrows indicate cells shown in the top right corner of images **c**, **f**, **i** at about 10 times magnification

**Fig. 3:**
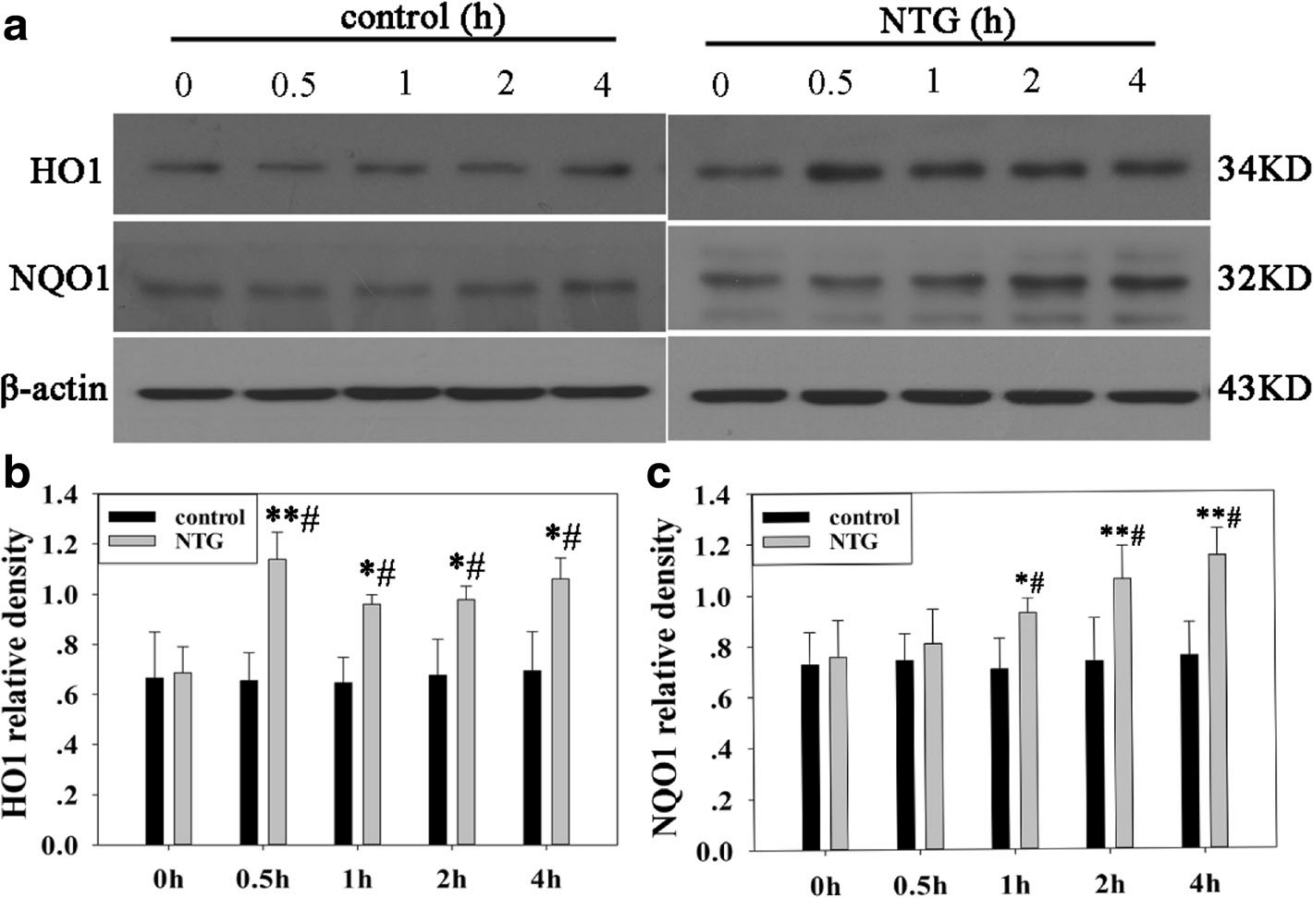
Effect of NTG injection on the levels of Nrf2 downstream proteins HO1 and NQO1 in rat TNC. **a** Representative immunoblots of TNC. Compared to the NS group or NTG 0 h group, HO1 levels were elevated at 0.5 h, 1 h, 2 h, and 4 h after NTG injection **b** and NQO1 levels were elevated at 1 h, 2 h, and 4 h after NTG injection **c**. β-actin was used as the loading control. Data are presented as relative density units normalized to β-actin, and expressed as mean ± SD. (* *P* < 0.05 *vs* the control group, **P < 0.01 *vs* the control group, # *P* < 0.01 *vs* NTG 0 h group, *n* = 6 per group)

To investigate the changes in the antioxidant system in rats treated with NTG, we analyzed Nrf2 expression in the nuclear and total cell fractions of TNC from rat models (Fig. 1). The subcutaneous administration of NTG (10 mg/kg) significantly increased Nrf2 levels in the total cell and nuclear fractions. This increase began within 0.5 h and persisted for 4 h after NTG injection. The control group with NS injection showed no statistical difference at the different time points. Moreover, immunofluorescence analysis (Fig. 2) showed that Nrf2 was located only in the neuronal cytoplasm of control group. Whereas, both nucleus and cytoplasm of neurons in the NTG group shared an obvious Nrf2 existence. We further analyzed the protein levels of two typical Nrf2-regulated phase II enzymes, HO1 and NQO1, in TNC of the rat models (Fig. 3). The expression of these two proteins also increased within 0.5 or 1 h, and persisted for 4 h after NTG exposure.

### Sulforaphane increased the expression of Nrf2 and the downstream proteins

**Fig. 4:**
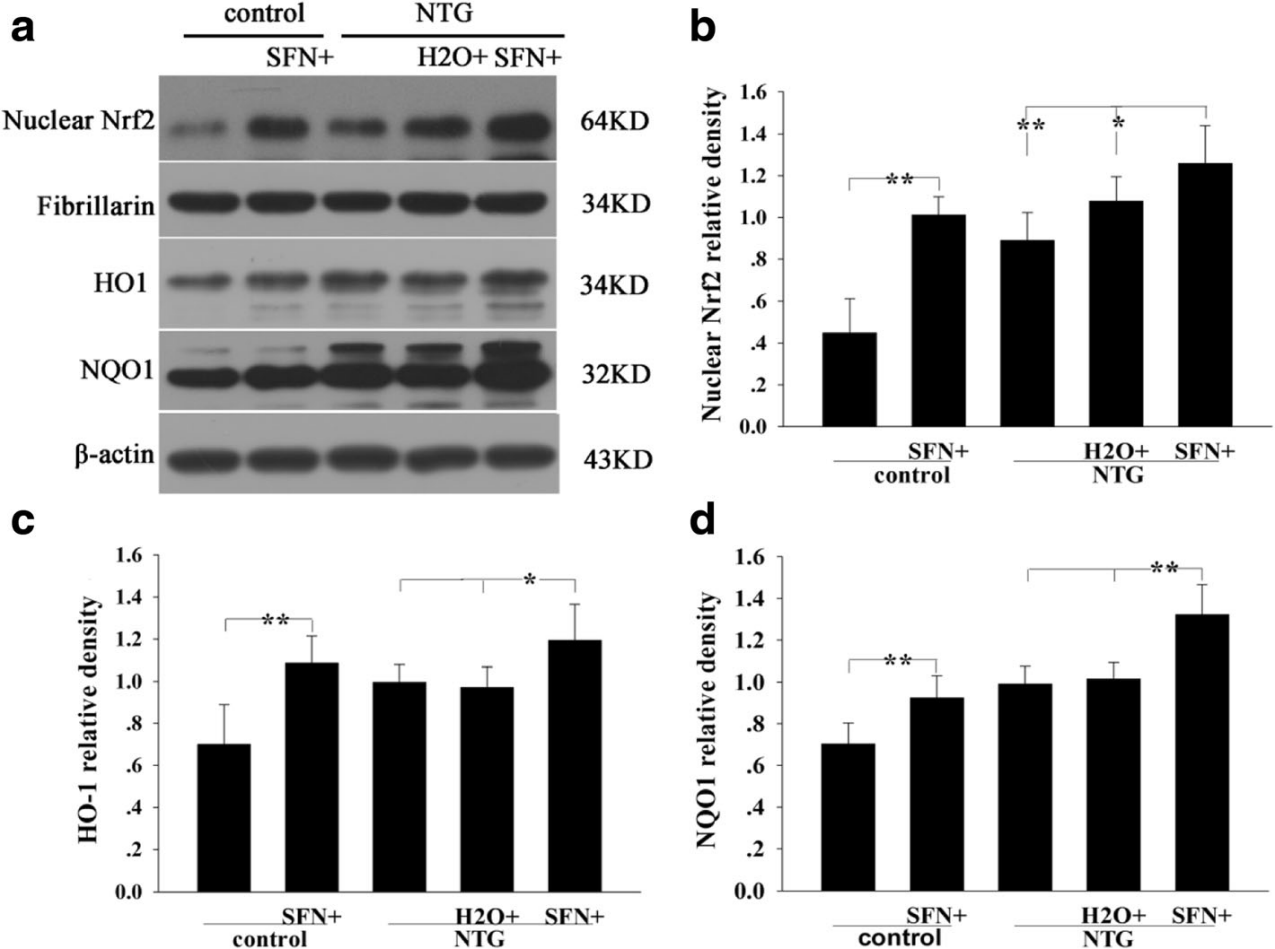
Effect of sulforaphane (SFN) on the levels of Nrf2 and downstream proteins HO1 and NQO1 in rat TNC 4 h after NTG injection. **a** Representative immunoblots of TNC. Nuclear Nrf2 **b**, HO1 **c** and NQO1 **d** levels were significantly increased in the SFN plus NTG group compared to those in the H_2_O plus NTG group. Moreover, these protein expressions were increased in the SFN plus control group compared to those in the control group **b**, **c**, **d**. Data are presented as the mean ± SD. (* *P* < 0.05, ** *P* < 0.01, *n* = 6 per group)

To determine the possible mechanism of Nrf2 effect, we used sulforaphane, a small-molecule inducer of this factor, to regulate the Nrf2/ARE pathway in this study. We performed immunoblotting to analyze the nuclear Nrf2, HO1, and NQO1 expressions. As shown in Fig. 4, sulforaphane treatment significantly increased nuclear Nrf2 level in NTG-treated rats (Fig. 4b). Moreover, it resulted in a significant increase in HO1 and NQO1 levels in these animals, compared to those in the control samples (Fig. 4c and d). Meanwhile, these protein expression levels were also increased in the sulforaphane plus control group compared to those in the control group.

### Sulforaphane pretreatment inhibited NTG-induced TGVS activation

**Fig. 5:**
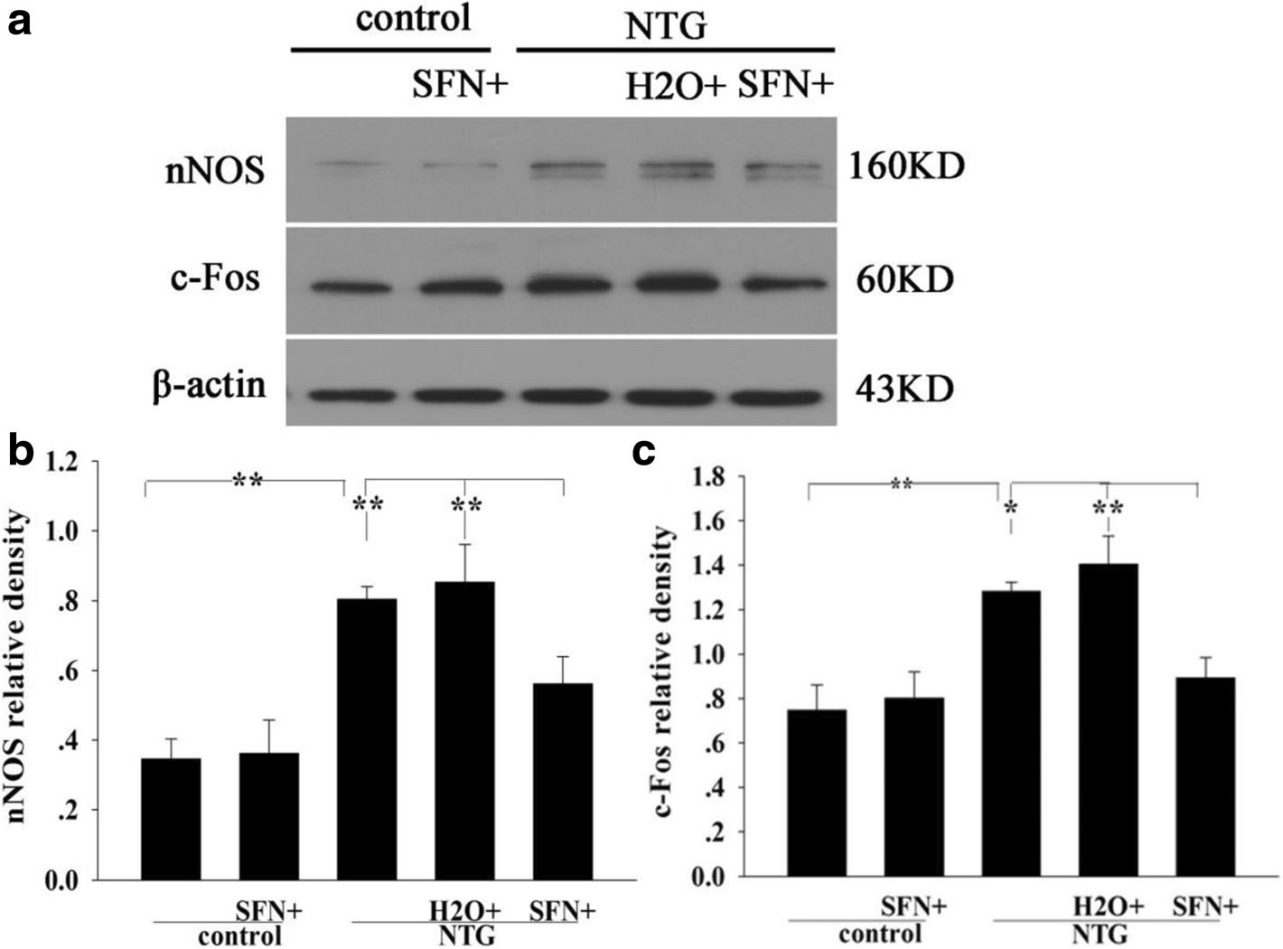
Effect of sulforaphane pretreatment on the levels of proteins nNOS and c-Fos in rat TNC 4 h after NTG injection. **a** Representative immunoblots of TNC. nNOS **b** and c-Fos **c** levels were significantly increased in the NTG group compared to those in the control group, decreased in the SFN plus NTG group compared to those in the H_2_O plus NTG group. Data are presented as the mean ± SD. (* *P* < 0.05, ** *P* < 0.01, *n* = 6 per group)

**Fig. 6:**
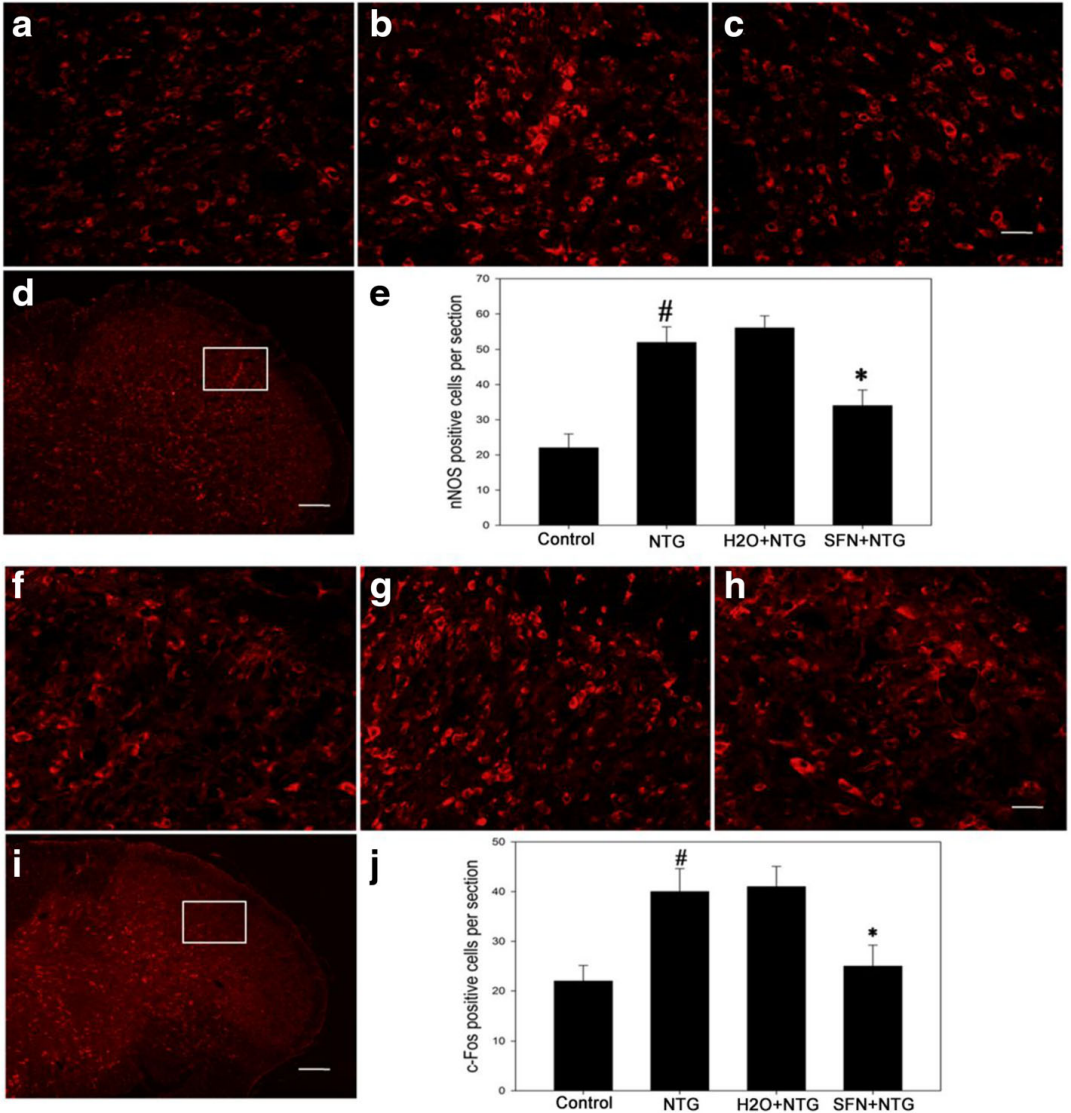
Effect of sulforaphane pretreatment on the number of nNOS- and c-Fos-immunoreactive cells in rat TNC 4 h after NTG injection. Panels a-d correspond to nNOS immunofluorescence staining, panels f-i correspond to c-Fos immunofluorescence staining. **a**,**f** control group. **b**,**g** NTG group. **c**,**h** SFN + NTG group. **d**,**i** the white rectangle frame indicates the representative anatomical site observed. Quantitative analysis showed that nNOS-immunoreactive **e** and c-Fos-immunoreactive cells **j** were all increased in the NTG group compared to those in the control group, but decreased in the SFN plus NTG group compared to those in the H_2_O plus NTG group. (* *P* < 0.05 for SFN + NTG *vs* H_2_O + NTG group, # *P* < 0.05 for NTG *vs* control group, *n* = 6 per group) (**a**, **b**, **c**, **f**, **g**, **h**: Bar = 50 μm; d, i: Bar = 200 μm)

Increased neuronal nitric oxide synthase (nNOS) and pro-oncogene c-Fos expressions in TNC are considered as the markers of TGVS activation [19, 20]. To check whether Nrf2 was involved in NTG-induced TGVS activation, we measured the expression levels of nNOS and c-Fos in NTG-treated samples at 4 h after sulforaphane pretreatment. As shown in Fig. 5, sulforaphane significantly reversed NTG-induced increase in nNOS and c-Fos expression. It also significantly reduced the number of nNOS- and c-Fos-immunoreactive neurons in TNC (Fig. 6).

### Sulforaphane pretreatment alleviated the NTG-induced cutaneous hyperalgesia

**Fig. 7:**
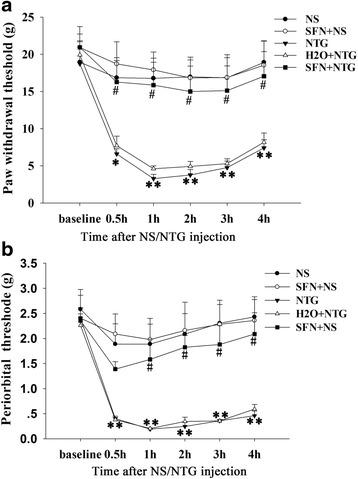
Effect of sulforaphane pretreatment on front paw withdrawal and periorbital thresholds in NTG-induced rats. **a** The front paw withdrawal thresholds significantly decreased as early as 0.5 h and persisted for 4 h in the NTG group compared to those in the control group, whereas the thresholds significantly increased as early as 0.5 h and persisted for 4 h in the SFN plus NTG group compared to those in the H_2_O plus NTG group. **b** The periorbital withdrawal thresholds also significantly decreased as early as 0.5 h and persisted for 4 h in the NTG group, while the thresholds significantly increased as early as 1 h and persisted for 4 h in the SFN plus NTG group compared to those in the H_2_O plus NTG group. (*P < 0.05, **P < 0.01 for NTG *vs* control group, ^#^ *P* < 0.05 for SFN + NTG *vs* H_2_O + NTG group. *n* = 6 per group)

We further investigated the efficacy of sulforaphane pretreatment on tactile sensitivity with calibrated von Frey hairs. We observed that subcutaneous administration of NTG significantly reduced the periorbital sensory and front paw withdrawal thresholds compared to that seen in the control group, which began 0.5 h after NTG injection (Fig. 7). Withdrawal thresholds of the front paw were significantly elevated in sulforaphane-pretreatment rats from 0.5–4 h after NTG injection (Fig. 7a). For periorbital tactile threshold analysis, an obvious increase was observed in the sulforaphane group from 1–4 h after NTG injection, as compared to the vehicle group (Fig. 7b).

## Discussion

Our study showed that the Nrf2/ARE signaling pathway in TNC was activated during NTG-induced migraine in rats. Sulforaphane pretreatment enhanced Nrf2 activation, increased the expression of HO1 and NQO1, decreased the expression of nNOS and c-Fos, and alleviated the NTG-induced hyperalgesia. These results indicated that oxidative stress was involved in NTG-induced hyperalgesia. Antioxidants may alleviate hyperalgesia via the suppression of TGVS activation. This study showed for the first time that sulforaphane, a natural Nrf2 activator compound, plays a protective role in NTG-induced hyperalgesia.

Under normal conditions, Nrf2 existence remains in the cytosol. Oxidative stressors can cause Nrf2 to translocate to the nucleus, thereby activating the Nrf2 pathway [21]. In this study, we observed that subcutaneous administration of NTG significantly increased nuclear Nrf2 expression in rat TNC. The levels of the two typical Nrf2-regulated phase II enzymes, HO1 and NQO1, were also increased. These data indicate that NTG induces oxidative stress, which contributes to the activation of Nrf2/ARE pathway. Moreover, NTG-induced oxidative stress has been proved to be involved in migraine pathogenesis [22, 23]. Thus, we believe that the Nrf2/ARE pathway is an endogenous adaptive compensatory factor in migraine. Similar to the situation in NTG-treated changes, Nrf2/ARE pathway is also activated in ischemic stroke, traumatic brain injury, and subarachnoid hemorrhage, and it shows compensatory adaptation [24–26]. Despite these observations, the mechanism of Nrf2/ARE pathway activation needs to be investigated further. NO induces Keap1 disulfide formation, Keap1 S-nitrosylation, or Keap1 S-guanylation. It can also induce oxidative or nitrosative stress which possibly induce the following activation of Nrf2, directly or indirectly through CRM1 or PI3K/PKC signaling pathway [27]. We also found that the total cellular Nrf2 expression was elevated. Consistent with the findings of the present study, inorganic arsenic induced an increase in Nrf2 protein by enhancing Nrf2 transcription [28]. We suggest that NTG may promote Nrf2 transcription, thereby increasing the Nrf2 protein level.

Activation of the Nrf2/ARE pathway is critical for neuroprotection [14, 29]. As an activator of Nrf2 pathway, sulforaphane is well known for its antioxidant and detoxification effects by inducing phase II genes [13]. We found in this study that sulforaphane activated Nrf2, upregulated downstream HO1 and NQO1, suppressed TGVS activation, and ameliorated the decrease of tactile thresholds in NTG-induced rats. These findings indicated that sulforaphane was probably involved in anti-hyperalgesia through the anti-oxidative stress Nrf2/ARE pathway. In addition, previous studies have shown that Nrf2-/- cells exhibit increased nNOS and c-Fos expression and oxidative damage [30, 31]. These data were similar to our findings of the suppressive effects of Nrf2 on nNOS and c-Fos. It is believed that activation of Nrf2 would inhibit TGVS ability by down-regulating nNOS and c-Fos expression. The above mechanisms may partly account for the efficacy of sulforaphane in migraine treatment. Moreover, anti-hyperalgesia efficacy of sulforaphane suggests that this compound reduces the production of proinflammatory cytokines and inhibits microglia activation [32–34]. These anti-inflammatory effects of sulforaphane may also contribute to its role in migraine treatment.

The present study shows that sulforaphane plays a therapeutic role in migraine solely via TNC neuronal activation. In future studies, Nrf2 gene knockout rats could be used to investigate the protective effect of sulforaphane. The underlying mechanism of sulforaphane action on migraine needs further investigation.

## Conclusions

In summary, our study initially demonstrated a critical role of the Nrf2/ARE signaling pathway in NTG-induced hyperalgesia rats. Our findings indicated that an Nrf2 activator, sulforaphane, inhibited the trigeminovascular system activation and prevented the induction of hyperalgesia, which provides a novel insight into the potential application of antioxidants as novel candidates in drug development for migraine.

## Abbreviations

i.p: intraperitoneally injection
NeuN: Neuronal nuclei
nNOS: Neuronal nitric oxide synthase
NO: Nitric oxide
Nrf2: Nuclear factor E2-related factor 2
Nrf2/ARE: The nuclear factor E2-related factor 2/antioxidant response element
NS: Normal saline
NTG: Nitroglycerin
PBS: Phosphate buffered saline
ROS: Reactive oxygen species
s.c: subcutaneous injection
SFN: R,S-Sulforaphane
TGVS: Trigeminovascular system
TNC: Trigeminal nucleus caudalis
